# Rehabilitation of aphasia: application of melodic-rhythmic therapy to Italian language

**DOI:** 10.3389/fnhum.2015.00520

**Published:** 2015-09-24

**Authors:** Maria Daniela Cortese, Francesco Riganello, Francesco Arcuri, Luigina Maria Pignataro, Iolanda Buglione

**Affiliations:** ^1^Intensive Care Unit, S. Anna Institute and Research in Advanced NeurorehabilitationCrotone, Italy; ^2^Casa di Cura Villa Margherita, San Giuseppe Moscati InstituteBenevento, Italy

**Keywords:** melodic intonation therapy, melodic rhythmic therapy, aphasia, broca, music therapy

## Abstract

Aphasia is a complex disorder, frequent after stroke (with an incidence of 38%), with a detailed pathophysiological characterization. Effective approaches are crucial for devising an efficient rehabilitative strategy, in order to address the everyday life and professional disability. Several rehabilitative procedures are based on psycholinguistic, cognitive, psychosocial or pragmatic approaches, including amongst those with a neurobehavioral approach the Melodic Intonation Therapy (MIT). Van Eeckhout’s adaptation of MIT to French language (Melodic-Rhythmic Therapy: MRT) has implemented the training strategy by adding a rhythmic structure reproducing French prosody. The purpose of this study was to adapt MRT rehabilitation procedures to Italian language and to verify its efficacy in a group of six chronic patients (five males) with severe non-fluent aphasia and without specific aphasic treatments during the previous 9 months. The patients were treated 4 days a week for 16 weeks, with sessions of 30–40 min. They were assessed 6 months after the end of the treatment (follow-up). The patients showed a significant improvement at the Aachener Aphasie Test (AAT) in different fields of spontaneous speech, with superimposable results at the follow-up. Albeit preliminary, these findings support the use of MRT in the rehabilitation after stroke. Specifically, MRT seems to benefit from its stronger structure than the available stimulation-facilitation procedures and allows a better quantification of the rehabilitation efficacy.

## Introduction

A frequent event after stroke is aphasia (with an incidence of 38% of cases; Pedersen et al., 1997; Engelter et al., 2006), which is multifaceted because of the brain structural and functional processes dedicated to, or involved in language (Mesulam, 1990; Bachman and Albert, 1991; Bookheimer, 2002; Démonet et al., 2005; Jung-Beeman, 2005). Linguistic and non-linguistic processes (*e.g.,* attention, memory, sensory or motor subroutines) are functionally related and their damage results in language impairment at varying levels of complexity. Accordingly, aphasia is a complex disorder (Huber et al., 1997; McNeil and Pratt, 2001); detailed pathophysiological characterization and proper approaches are mandatory for an efficient rehabilitative strategy to be devised and the disability in everyday life and profession to be compensated for Black-Schaffer and Osberg (1990), Holland et al. (1996), Paolucci et al. (1997), Robey (1998) and Tilling et al. (2001).

Some of the most common varieties of aphasia are classified in two major forms: fluent and non-fluent. The fluent form is generally characterized by the impairment to grasp the meaning of spoken words, while the ease of producing connected speech is not affected so critically. Therefore Wernicke’s aphasia is referred to as a “fluent aphasia”. However, speech is far from normal. Sentences do not hang together and irrelevant words intrude sometimes to the point of jargon, in severe cases. Reading and writing are often severely impaired (Stringer and Green, 1996).

The second form of aphasia is characterized by severe reduction of speech output, limited mainly to short utterances of less than four words. Vocabulary access is limited and the formation of sounds by persons with Broca’s aphasia is often laborious and clumsy. The person may understand speech relatively well and be able to read, but be limited in writing (Stringer and Green, 1996). Broca’s aphasia is often referred to as a “non fluent aphasia”, characterized by anomia (i.e., word-retrieval difficulty), agrammatism (i.e., grammar and syntax deficit), and apraxia of speech (AOS; a motor speech disorder affecting the planning or programming of speech movements; American Academy of Neurology, 1994; Ballard et al., 2000). However if anomia is the core symptom of aphasia, and is present in all aphasic syndromes, agrammatism and AOS are clinical markers used to differentiate Broca’s from other aphasias. MIT (Albert et al., 1973) has shown little effect on agrammatism. More, the hypothesis that MIT could be effective on Broca’s aphasia is due to its action on deficit in motor planning or programming of speech movements.

No indications are referred to the application of MIT to global aphasia patients, characterized by the production of no or few recognizable words and no or poor comprehension of spoken language. Moreover, global aphasia patients can neither read nor write (Stringer and Green, 1996).

There is widespread consensus on the efficacy of the different rehabilitative approaches of the aphasic (Brain Injury Interdisciplinary Special Interest Group; American Congress of Rehabilitation Medicine; European Federation of Neurological Societies; Cappa et al., 2005; Ciceron et al., 2011). However, efficacy can vary among subjects. Variability depends on the applied rehabilitative procedure as well as on the intensity of treatment (Brindley et al., 1989; Poeck et al., 1989; Teasell et al., 2003), with better recovery after intensive and prolonged rehabilitation (Bhogal et al., 2003). Several rehabilitative procedures are available based on psycholinguistic (Schwartz and Fink, 1997; Lesser and Milroy, 2014), cognitive (Holland, 1994), psychosocial or pragmatic (Holland, 1991; Lyon et al., 1997; Elman, 1998) approaches. However, major limitations of, and source of criticism to the rehabilitation of the aphasic, rest on the inadequate tailoring of rehabilitative procedures to the individual patient’s needs.

Among the rehabilitative procedures with neurobehavioral rationale, the Melodic Intonation Therapy (MIT) designed by Albert and co-workers (Table 1; Albert et al., 1973; Sparks et al., 1974; Sparks and Holland, 1976) has been rated *promising* (class III) by the Therapeutics and Technology Assessment Subcommittee of the American Academy of Neurology (American Academy of Neurology, 1994) and transferred with comparable results to non-English linguistic populations such as Romanian, Persian, and Japanese (Seki and Sugishita, 1983; Popovici and Mihailescu, 1992; Baker, 2000; Bonakdarpour et al., 2003). Van Eeckhout ‘s adaptation to French language (Melodic-Rhythmic Therapy, MRT; Van Eeckhout and Bhatt, 1984) has implemented the training strategy by adding a rhythmic structure reproducing French prosody, i.e., with melodic interval of 4th and the presentation of sentences in rhythmic epochs matching the syntactic-semantic structure (Tranel, 1987; Rossi, 1999; Hind, 2002; Van Eeckhout, 2002). The approach proved efficient and suggests application to other languages. Purposes of this study were to adapt MRT rehabilitation procedures to Italian language and to verify its efficacy in a group of patients with severe non-fluent aphasia.

**Table 1:** **Melodic intonation therapy (MIT)**.

Level I	Level II	Level III	Level IV
Presentation of short item (max five syllables) with an arbitrary melody. The patients repeat the melody (tapping it out) with the therapist’s assistance decreasing over time.	Presentation of four-note melodies, with larger tonal interval and increasing length of words and sentences.	Global repetition with minimal or no therapist’s assistance. Sentences are longer and the syntactic-semantic structure complexity increases. Introduction of the *Sprechgesang*.	The method incorporates *sprechgesang* at this level. More complex phrases and longer sentences are attempted.

*MIT is a “formal” treatment with hierarchical structure developed in four levels, assuming a key role of the right hemisphere in the control of the speech accent, intonation and melodic pattern*.

## Materials and Methods

### Adaptation

Rhythmic-temporal and melodic-intonative plans are considered the characteristic features of prosodic aspects, or suprasegmental, of language. In particular, in the speech, the rhythm refers to the prominent elements and not the phonetic string, while the tone refers to the pitch and loudness variations (Marotta, 2009). The basic unit of rhythm is the syllable (phonetically and phonologically defined as an agglomeration of phonic elements around an intensity or loudness peak) and the alternation of strong and weak syllables is the analysis and the creation of a rhythmic pattern. The prominence, or force, is determined by the accent, i.e., an increase of intensity, duration and height, with respect to the adjacent elements (Savy, 2009).

Depending on the characteristics of the rhythm, natural languages have been divided into stress-timed (with regular intervals between the accents) and syllable-timed, (with constant syllabic duration), as Italian. In addition, there are other elements that allow classifying languages, such as the compressibility of unstressed syllables, defined “compensation languages”, e.g., English. Other languages do not allow it, they are defined “check languages”, e.g., Italian (Romito and Trumper, 1993).

Among the models developed to analyze the rhythmic characteristics of the languages, a recent one, the Control/Compensation Index, is able to create groupings, in order to define the belonging of a language to the syllable-or stress-timed group (Bertinetto and Bertini, 2008). Unlike other prosodic elements, the tone is intrinsically significant. Even Italian, although it is not a tonal language (such as Mandarin), can be represented as a sequence of two types of discrete tones: High and Low, e.g., a decreasing tone characterizes a “declaration”, and an increasing tone characterizes a “question” (Bertini and Bertinetto, 2007).

**Figure 1:**
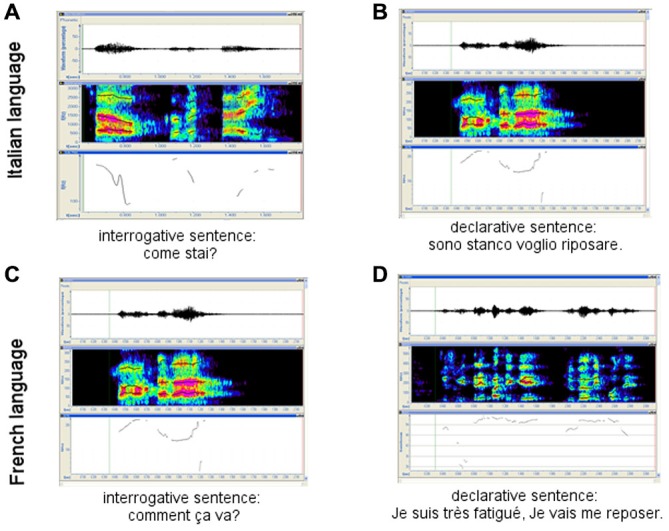
**French and Italian interrogative vs. declarative sentences. (A)** and **(C)** interrogative sentences in Italian and French Language respectively; **(B)** and **(D)** declarative sentences in Italian and French Language respectively.

**Figure 2:**
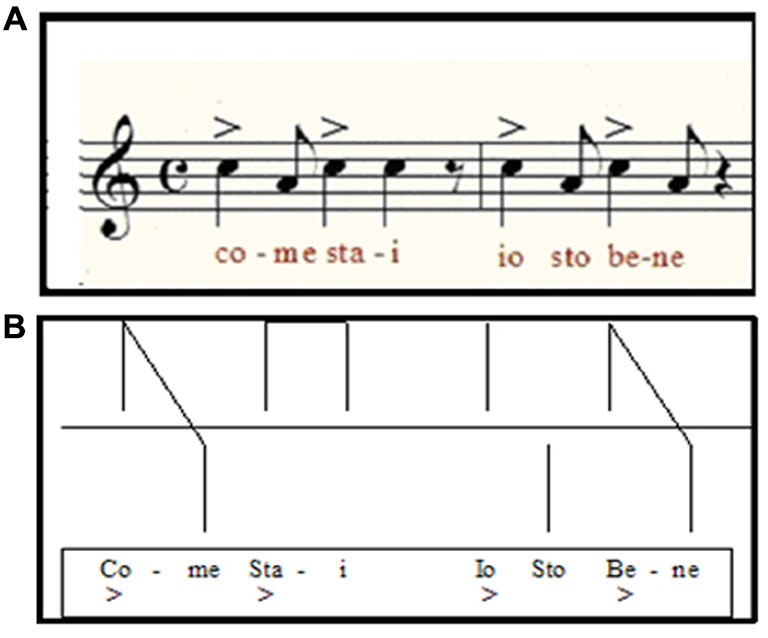
**(A)** Example of the melodic-rhythmic structure of an Italian sentence of common use (“how are you? I am fine”) and its visual scheme used in MRT **(B)**.

A peculiarity of MRT in French version is the use of a core melodic sequence based on two notes (high and low, respectively), with stressed accentuation and slow scanned rhythm (Van Eeckhout et al., 1995; Table 2; Figures 1C,D). French language is characterized by a consonant at the end of most words, a tonic accent falling most often on the last vowel, a high prolonged note at the beginning of most sentences, and the sentence subdivision in syntactic-semantic units (Tables 3, 4). Instead, Italian language is organized in “tonal units” (Hart et al., 2006) that contribute to the sentence rhythmic scan and also add in the communication of meaning (Cresti, 1987). MRT adaptation to Italian language was therefore performed by adjusting to the tone and prosody properties of this language (Tables 3, 4; Figures 1, 2A,B) and by taking in due consideration the role of these properties in spontaneous linguistic communication (Chapallaz, 1964; Austin, 1975; Bertinetto, 1981; Vayra and Fowler, 1987; De Dominicis and Vineis, 1992; Bertinetto and Magno Caldognetto, 1993; Savy et al., 2004). To this end, the tonal interval of 3rd major has been selected (Romano, 2001; Romano and Interlandi, 2005), with the high and low notes positioned where the tonic accent falls and a low note at the end of the sentence (with the exception of words with the last syllable stressed or interrogative sentences).

**Table 2:** **MRT parameters**.

Melody	Two different notes, defined by pitch, intensity and duration: one note acute and prolonged (*e.g.,*semibreve) and the other grave and short (*e.g.,* chrome) with a tonal interval of 3rd mayor.
Rhythm	Accentuation of selected syllables when “singing” the sentence.
Scanning	Tapping of rhythm, by the hand of the homolateral side to the injury, in order to promote a “contact” between patient and therapist.
Accentuation	Accentuation of omitted sections of the sentence that are instead indicated by visual symbols.
Visual scheme	Graphic representation of the sentence in function of melody.

**Table 3:** **Intonation/prosody peculiarities of Italian language**.

–	Tonal elevation on the first part of the sentence and usually descendent intonation, with a stable or further descending profile at the end of sentence (on the tonic or eventually post-tonic syllable; Figure 1B).
–	Descending-ascending profile at the end of questions (Figure 1A).
–	Accented vowels longer than atone vowels.
–	Phonetic reduction (at least in the pitch) of atone vowels.
–	Pitch reduction of atone vowels peculiar to the phonetic Italian accent, traditionally identified as a language with syllabic isochrony.

**Table 4:** **French and Italian linguistic features**.

	Linguistic features		MRT—Italian vs. French					
	Question profile	Tonic accent	Tone interval	Start of the sentence	Tonic syllable	End of sentence with acute note	End of sentence with bass note	Syntactic-semantic subdivision of the sentence
Italian language	Ascending-descending course at the last part of the sentence	Relatively free	Third mayor	Acute and prolonged note	Acute and prolonged note	Interrogative sentence or words with the accent in the last syllable	Declarative sentence and sentence without the accent on the last syllable	Yes
French language	Ascending-descending course at the final part of the sentence	Primarily it falls on the last syllable	Fourth	Acute and prolonged note	Acute and prolonged note	Always	Never	Yes

### Patients

Six patients (five males) with ischemic stroke in dominant hemisphere and non-fluent Broca’s aphasia rated severe at the Aachener Aphasie Test (AAT; Huber et al., 1983; Luzzatti et al., 1996) were admitted to the study at least 9 months after brain injury. Age was 59.8 ± 9.3 years (range: 53–71 years); education ranged from grammar school to university. In all cases, brain damage was unilateral; spontaneous speech, word articulation and repetition of single words were impaired; comprehension of spoken language was maintained; acoustic perception was documented across a wide range of sound frequencies; patients were motivated and emotionally stable. All subjects had been treated by conventional speech therapy rehabilitation procedures for 3–17 months before entering the study. Upon admission to the study, they underwent a baseline standard assessment of their residual language performance (AAT), that was proved to be superimposable to the last evaluation, referred to the end of the traditional treatment. The sample size and follow-up evaluation met the requirements of the “American Academy of Neurology, Therapeutics and Technology Assessment Subcommittee”. The study has been approved by the local public health care Ethical Committee. Subjects were informed in full detail about the study purpose and experimental procedures, more codes and policies for research (Resnik, 2011) of the ethical principles of the Declaration of Helsinki (1964) by the World Medical Association concerning human experimentation were followed.

### MRT Rehabilitation Protocol

Patients were treated intensively (Schlaug et al., 2009; Wan et al., 2014) 4 days a week for 16 weeks. Each session lasted 30–40 min; apraxia was treated for about 10 min at the beginning of the rehabilitation session. In all cases, the following procedures were applied in hierarchical sequence:

1. Non-verbal rhythmic and melodic exercises for the patient to approach the methodological strategy through the relationship between the rhythmic-melodic sequence and the sentences to be reproduced. In this phase, the patient is also trained to perform without a direct visual contact with the therapist.
2. Patients were requested to repeat with rhythmic-melodic scan 25 common sentences of increasing complexity and length (e.g., I am fine; I wish to meet my friends, etc.). At this stage repetition was controlled by the therapist, whose help is progressively reduced with the patient improving repetition of all sentences by him/herself. Each sentence was attributed the score 1 (if adequately repeated and understandable) or zero. Sentences difficult to pronounce were presented again after focusing on the problem. Upgrading to phase 3 was allowed when 90% of sentences were properly reproduced without the therapist’s help and visual contact.
3. In the third phase, sentences were sorted out of the patient’s daily life (life in hospital, news, etc.) and the subjects were forced to use the rhythmic-melodic scan to communicate (Table 5).

**Table 5:** **MRT phases**.

**Phase I: nonverbal training**
1 Reproduction of rhythmic sequences	Simple sentences such as questions-answers (e.g., how are you?/I am fine) in rhythmic form.	16 sequence with increasing length and complexity.
2 Rhythmic conversation	Reproduction of 10 melodic sequences of increasing length.	
3 Humming	
4 Reading of melodic patterns
**Phase II: full set of 25 sentences with increasing length and complexity**
1 Intonation		The therapist gives the sentence tone while marking the rhythm on the patient’s hand.
2 Unison	The therapist and patients intone the sentences together.	
3 Unison with progressively reduced therapist’s assistance	The therapist begins the sentence and the patient is requested to conclude it.
4 Immediate repetition	The therapist intones the sentence while the rhythm on the patient’s hand and request immediate repetition.
5 Therapist asks questions and each answer is used in the conditions outlined above	
**Phase III**
Same steps as in Phase II, but with sentences picked up from the patient’s everyday’s life

### Follow-Up

The efficacy of MRT was assessed by means of the AAT both at the end of rehabilitation and 6 months later.

### Data Analysis

The differences between baseline and end of the treatment, as well as the difference between end of treatment and control at follow-up were tested statistically by the Wilcoxon’s exact test (Siegel, 1956; Gibbons and Chakraborti, 2011), that is more accurate in case of small sample, or when the tables are sparse or unbalanced (Tanizaki, 1997; Mundry and Fischer, 1998; Gibbons and Chakraborti, 2011). The effect size (r; i.e., the index measuring the magnitude of difference or change between two conditions, in this case baseline vs. end of the protocol; Rosenthal, 1991) was calculated as the z/square root (N; where N is the number of observations on which z is based) and will be hereafter formally referred to as not relevant (*r* < 0.1), small (0.1 < *r* < 0.3), medium (0.3 < *r* < 0.5), or large (*r* > 0.5; Hemphill, 2003).

## Results

**Figure 3:**
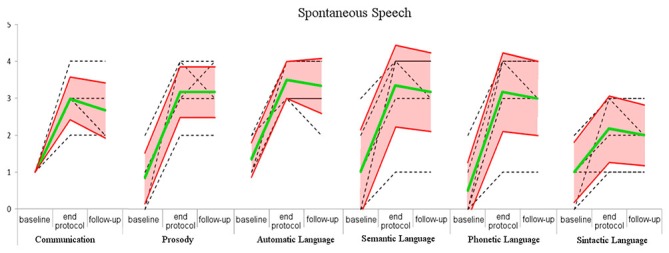
**Score of the spontaneous speech in baseline, end of the protocol and follow-up.** Green line: mean of the scores; red lines: standard deviation; black dashed line: raw data.

At baseline, the patients’ speech was restricted to few, fragmentary and scarcely understandable sentences. Anomies, agramatisms, phonemic paraphasias, neologisms and perseverations were observed as indicative of partial efficacy of conventional rehabilitation on spontaneous speech. Spontaneous speech (as measured by the AAT test; Figure 3; Table 6) was improved at the end of MRT specifically, in the semantic-lexical structure (Wilcoxon exact test: *z* = −2.220, *p* = 0.031, *r* = 0.640), phonemic structure (Wilcoxon exact test: *z* = −2.226, *p* = 0.031, *r* = 0.642), speech automatism (Wilcoxon exact test: *z* = −2.332, *p* = 0.031, *r* = 0.673), prosody (Wilcoxon exact test: *z* = −2.333, *p* = 0.031, *r* = 0.673) and communication (Wilcoxon exact test: *z* = −2.264, *p* = 0.031, *r* = 0.653). Moreover improvements were found in the correct repetition (Wilcoxon exact test: *z* = −2.207, *p* = 0.031, *r* = 0.637), naming (Wilcoxon exact test: *z* = −2.201, *p* = 0.031, *r* = 0.635), and comprehension (Wilcoxon exact test: *z* = −2.201, *p* = 0.031, *r* = 0.635) subtests (Figure 3; Table 7). The number of pronounced words per interval time increased, phonemic structure and syntax improved too. At follow-up, the AAT ratings in all subtests were superimposable to those recorded at the end of rehabilitation in the spontaneous speech as well as in the subtest (z ≤ −1.633, *p* ≥ 0.125).

**Table 6:** **AAT speech language**.

	Communication			Prosody			Automatic language			Semantic language			Phonetic language			Syntactic language		
Patient	Start	End	Follow-up	Start	End	Follow-up	Start	End	Follow-up	Start	End	follow-up	Start	End	Follow-up	Start	End	Follow-up
1	1	3	3	1	3	3	1	4	4	0	4	4	1	4	4	0	3	3
2	1	2	2	2	4	4	2	3	3	3	4	4	2	4	4	2	3	3
3	1	3	3	0	4	3	1	3	3	0	1	1	0	4	3	0	1	1
4	1	3	2	0	2	2	1	4	4	0	4	3	0	3	3	1	2	2
5	1	4	4	1	3	4	2	4	4	2	4	4	0	3	3	2	3	2
6	1	3	2	1	3	3	1	3	2	1	3	3	0	1	1	1	1	1

**Table 7:** **AAT sub-test**.

	Token			Repetition			Writing			Denomination			Comprehension		
Patient	Start	End	Follow-up	Start	End	Follow-up	Start	End	Follow-up	Start	End	Follow-up	Start	End	Follow-up
1	43	11	11	44	46	48	0	15	15	0	18	18	73	95	96
2	16	14	12	33	50	50	1	27	25	25	26	28	92	98	99
3	43	38	38	33	43	43	0	3	3	0	5	4	40	58	58
4	44	40	38	38	42	42	0	4	4	19	21	21	50	67	67
5	36	36	36	30	32	31	2	10	9	16	38	40	85	97	99
6	37	33	33	44	60	60	3	10	10	5	14	14	74	85	85

## Discussion

The emerging research field of music and neuroscience has evidenced that the sound envelope processing (Kotz and Schwartze, 2010; Patel, 2011; Peelle and Davis, 2012) and the synchronization and entrainment to a pulse, may help to stimulate brain networks for human communication (Fujii and Wan, 2014). The possible circuits that may help to stimulate the brain networks underlying human communication could be: (1) the auditory afferent circuit consisted of brainstem, thalamus, cerebellum, and temporal cortex for precise encoding of sound envelope and temporal events (Kotz and Schwartze, 2010); (2) the subcortical–prefrontal circuit for emotional and reward-related processing (Koelsch, 2014); (3) the basal ganglia-thalamo-cortical circuit for processing beat-based timing (Kotz and Schwartze, 2011); and (4) the cortical motor efferent circuit for motor output (Meister et al., 2009a,b).

The cortical functional re-organization underlying recovery of language remains poorly understood. PET or fMRI studies have related the recovery of impaired language with increased activation of cortical area of the right hemisphere involved in language (Cappa et al., 1997; Thulborn et al., 1999; Gold and Kertesz, 2000) the result of inadequate compensation processes for others (Rosen et al., 2000; Perani et al., 2003; Naeser et al., 2004), while other studies indicate activation of peri-lesional areas in the left hemisphere as the key mechanism for an efficient recovery to occur (Karbe et al., 1998; Cao et al., 1999; Heiss et al., 1999). PET studies on aphasic patients with no spontaneous recovery undergoing MIT rehabilitation (Belin et al., 1996; Warburton et al., 1999) have documented activation of left Broca’s area and concomitant inhibition of contralateral Wernike’s area. Compensatory re-activation in response to MIT/MRT rehabilitation of the left hemisphere structures involved in language (e.g., Heschl’s gyrus, temporal pole, angular gyrus, Broca’s area and adjacent prefrontal cortex) therefore is a practicable hypothesis. Further investigation is required to correlate the efficacy of MRT rehabilitation with the extent of brain damage at baseline and changes in the brain functional organization as documentable e.g., by advanced neuroimaging techniques.

## Conclusion

Use of MRT in neo-latin countries required adjustment to the language metrics. Adaptation to Italian language rhythm and prosody (Pöchhacker, 1994; Hart et al., 2006) according to metrics criteria (Tables 3, 4) proved successful: impaired speech improved in our chronic patients’ sample. Albeit preliminary, these findings support the use of MRT in the rehabilitation after stroke. Specifically, MRT seems to benefit from its stronger structure than the available stimulation-facilitation procedures and allows a better quantification of the rehabilitation efficacy. In this regard, it compensates in part for the current problems in documenting the individual patient’s improvement in his/her interacting environment (unit, family, everyday life) and appears more reliable than standard evaluation scales (AAT, BDAE etc.). The observation of improved written language suggests possible selective application in the treatment of this deficit and upgraded research on the mechanisms undergoing writing and its impairment in aphasia.

## Author Contributions

All authors were involved in the conception and design or analysis and interpretation of data, have contributed to the drafting and revisions of the manuscript, and have approved the submitted version.

## Conflict of Interest Statement

The authors declare that the research was conducted in the absence of any commercial or financial relationships that could be construed as a potential conflict of interest.
